# Cloud-Based Machine Learning Platform to Predict Clinical Outcomes at Home for Patients With Cardiovascular Conditions Discharged From Hospital: Clinical Trial

**DOI:** 10.2196/45130

**Published:** 2024-03-01

**Authors:** Phillip C Yang, Alokkumar Jha, William Xu, Zitao Song, Patrick Jamp, Jeffrey J Teuteberg

**Affiliations:** 1 Stanford University School of Medicine Palo Alto, CA United States; 2 AiCare Corporation San Jose, CA United States; 3 Emory University Atlanta, GA United States; 4 North Carolina State University Raleigh, NC United States; 5 Electrical Engineering University of California, Los Angeles Mountain View, CA United States

**Keywords:** smart sensor, wearable technology, moving average, physical activity, artificial intelligence, AI

## Abstract

**Background:**

Hospitalizations account for almost one-third of the US $4.1 trillion health care cost in the United States. A substantial portion of these hospitalizations are attributed to readmissions, which led to the establishment of the Hospital Readmissions Reduction Program (HRRP) in 2012. The HRRP reduces payments to hospitals with excess readmissions. In 2018, >US $700 million was withheld; this is expected to exceed US $1 billion by 2022. More importantly, there is nothing more physically and emotionally taxing for readmitted patients and demoralizing for hospital physicians, nurses, and administrators. Given this high uncertainty of proper home recovery, intelligent monitoring is needed to predict the outcome of discharged patients to reduce readmissions. Physical activity (PA) is one of the major determinants for overall clinical outcomes in diabetes, hypertension, hyperlipidemia, heart failure, cancer, and mental health issues. These are the exact comorbidities that increase readmission rates, underlining the importance of PA in assessing the recovery of patients by quantitative measurement beyond the questionnaire and survey methods.

**Objective:**

This study aims to develop a remote, low-cost, and cloud-based machine learning (ML) platform to enable the precision health monitoring of PA, which may fundamentally alter the delivery of home health care. To validate this technology, we conducted a clinical trial to test the ability of our platform to predict clinical outcomes in discharged patients.

**Methods:**

Our platform consists of a wearable device, which includes an accelerometer and a Bluetooth sensor, and an iPhone connected to our cloud-based ML interface to analyze PA remotely and predict clinical outcomes. This system was deployed at a skilled nursing facility where we collected >17,000 person-day data points over 2 years, generating a solid training database. We used these data to train our extreme gradient boosting (XGBoost)–based ML environment to conduct a clinical trial*, Activity Assessment of Patients Discharged from Hospital-I*, to test the hypothesis that a comprehensive profile of PA would predict clinical outcome. We developed an advanced data-driven analytic platform that predicts the clinical outcome based on accurate measurements of PA. Artificial intelligence or an ML algorithm was used to analyze the data to predict short-term health outcome.

**Results:**

We enrolled 52 patients discharged from Stanford Hospital. Our data demonstrated a robust predictive system to forecast health outcome in the enrolled patients based on their PA data. We achieved precise prediction of the patients’ clinical outcomes with a sensitivity of 87%, a specificity of 79%, and an accuracy of 85%.

**Conclusions:**

To date, there are no reliable clinical data, using a wearable device, regarding monitoring discharged patients to predict their recovery. We conducted a clinical trial to assess outcome data rigorously to be used reliably for remote home care by patients, health care professionals, and caretakers.

## Introduction

### Background

Why are some discharged patients readmitted whereas others are not? Although often routine and uncomplicated, this transition of care is complex and, if not arranged properly, can lead to life-threatening consequences. Clearly, factors such as disease severity and the intensity of postdischarge care affect the risk of readmission; however, many other issues may also have substantial contributions [1]. The top contributory factors are (1) admission diagnosis: heart failure is the top cause of readmission, whereas other conditions, including sepsis, pneumonia, chronic obstructive pulmonary disease, and cardiac arrhythmia, are considered high risk; (2) insurance: Medicare and Medicaid patients have the highest rates of readmission; (3) patient demographics: race, sex, age, and income play a key role (eg, women who experience heart attacks and populations with lower-income status); and (4) patient engagement: patients who lack knowledge, skills, and confidence to manage their care have nearly double the average readmission rate [2,3].

The hospital readmission rate is approximately 20% in the United States, and the rates increase proportionately among those who are aged ≥50 years [4]. Our health care infrastructure is overburdened. Therefore, it is incumbent upon health care providers to develop risk stratification algorithms expeditiously to help predict which patients are at the highest risk for readmission. However, this determination is extremely difficult to make, particularly because the majority of the discharged patients will not become critically ill, require readmission, or die. Therefore, as we try to mitigate the risks of readmission, we need to do more than just predict the risk of readmission; we need to also tailor our home monitoring strategies to this risk [1]. Furthermore, this monitoring must not overwhelm health care providers or patients; rather, the aim should be to deliver smart, robust, and intelligent monitoring of patients convalescing at home.

Physical activity (PA) is one of the major determinants for overall clinical outcomes in chronic diseases, including diabetes, hypertension, hyperlipidemia, heart failure, and cancer, as well as mental health issues [5-8]. Moreover, these same comorbidities increase the risk of readmission. In 2017, the Centers for Disease Control and Prevention advocated adding PA as the fourth vital sign after heart rate (HR), blood pressure, and body temperature [9]. These developments underline the critical importance of PA in assessing the recovery of patients and, more importantly, indicate a clear need to measure PA quantitatively beyond the current questionnaire and survey methods [4,9,10]. PA is defined simply as any bodily movement produced by the skeletal muscles that result in energy expenditure [11]. However, it has been difficult historically to directly measure PA. It requires a dedicated laboratory to measure and perform a kinematic analysis. In addition, the measurement period is short and hard to monitor over time. Wearable technology and wireless data transmission have overcome these limitations and facilitate an accessible and long-term assessment of PA. A triaxial movement sensor was found to be a reliable, valid, and stable measurement of walking and daily PA in patients with chronic obstructive pulmonary disease [7]. Furthermore, a portable system for PA assessment in a home environment has been proposed [5]. These innovative systems provide novel and comprehensive real-time data for the evaluation of the health and quality of life of participants with limited mobility and chronic diseases. Finally, an estimate of step counts and energy expenditure strongly correlated with observed step counts and measured energy expenditure, using hip- and wrist-based Fitbit devices [6].

### Development of an Advanced Data-Driven Analytic Platform

We developed an advanced data-driven analytic platform that predicts clinical outcomes based on accurate measurements of PA [10]. Artificial intelligence (AI) or machine learning (ML) analyzes the data to predict short- and long-term health outcomes. Although there is an overabundance of wearable devices (WDs) in the market, there are no known clinical outcome data that could be used reliably for home care by patients, health care professionals, or caretakers. In conjunction with AiCare Corp in San Jose, California, United States, we developed a platform consisting of the following key components: (1) a WD synced to an iPhone or app, (2) a web-based open application programming interface (API), (3) an AI and ML interface, and (4) a Health Insurance Portability and Accountability Act (HIPAA)–compliant Amazon Web Services (AWS) server environment. This platform was deployed at a skilled nursing facility where we collected >17,000 person-day data points (408,000 person-hour data points). These data provided the training set for our extreme gradient boosting (XGBoost) AI algorithm to correlate PA data to health outcomes in the *Activity Assessment of Patients Discharged from Hospital-I (ACT-I) clinical trial*. In this ACT-I trial, we enrolled 52 patients discharged from Stanford Hospital. Our data demonstrated a robust predictive system to forecast health outcomes in the enrolled patients based on their PA data. The clinical study generated a receiver operating characteristic (ROC) analysis with a sensitivity of 87% and a specificity of 79% in predicting the clinically significant events that were reported by the patients. Our comprehensive AI profiling of the PA of the discharged patients predicted their recovery or clinical deterioration to enable the precision guidance of appropriate and timely intervention during the 4-week follow-up period.

After considering various functionalities and requirements, the WD offered the most practical and compliant design solution to monitor discharged patients intelligently. However, the wearable technology for discharged patients should embody different applications and designs specific to their needs. In this paper, we will demonstrate these specifications in more detail. We will present AiCare’s comprehensive technology solution consisting of a WD, Bluetooth low energy (BLE)–enabled iOS infrastructure, an ML algorithm to implement AI in patient care, and API-enabled web technology to measure the daily activities of patients. In this study, remote data collection, robust XGBoost AI analysis, and the reliable prediction of clinical outcomes are reported.

## Methods

### Patient Recruitment

We screened patients discharged from Stanford Hospital general cardiology and advanced heart failure program.

### Ethical Considerations

We obtained approval from the Stanford University Institutional Review Board (53805) and recruited patients discharged from Stanford Hospital. They were invited to participate in a research study to demonstrate the safety and feasibility of the AiCare platform. Informed consent was obtained from each participant who consented to primary data collection and secondary data analysis without additional consent. The privacy and confidentiality of participants are protected by a deidentified code that is assigned to each patient. No compensation was offered to participants.

### ML Predictive Platform

Our comprehensive ML profile of the discharged patients was designed to predict their proper recovery to enable the precision guidance of timely intervention during the 4-week follow-up period. We developed an advanced data-driven XGBoost analytics platform to predict clinical outcomes based on accurate measurements of PA [10].

We compared several techniques to analyze our training data set, including logistic regression, naïve Bayes, support vector machine, and XGBoost. We measured precision, recall, *F*_1_-score, area under the ROC curve (AUC-ROC), and average critical activity level, using different data sets. Throughout the analyses, XGBoost provided the highest area under the curve (AUC) values and other measurements.

We chose XGBoost because of its interpretability through the model training process, resistance to trivial features, and the reduced risk of overfitting. For our health care use case, model transparency was an important evaluation criterion. XGBoost visualized the feature prioritization and automatic weight assignments, which allowed us to explain the model insights to the stakeholders for solution adoption. Occasionally, there are noises in sensor data. To reduce the risk of overfitting, we experimented with max_depth of 2, 3, 4, and 5 and min_child_weight of 1, 2, 3, 4, and 5. On the basis of our list of PA-related input feature set and data volume (>17,000 person-day data points), the hyperparameters we used were max_depth of 3, learning_rate of 0.01, min_child_weight of 4, and n_estimators of 100. This achieved the balance of model accuracy, reducing the risk of overfitting and reaching a tolerable learning speed. We experimented with both XGBoost 1.4.1 and XGBoost 1.7.5. A mean absolute error of 1.7.x was introduced, which boosted the training algorithm convergence process. Some assumptions we made regarding the XGBoost model that should be considered include that the encoded integer values for each input variable have an ordinal relationship; it should not be assumed that all values are present. Our algorithm could handle missing values by default. In our tree-based algorithm, missing values were learned during the training phase.

Our AI platform predicted clinical outcome risk during the 4-week follow-up period using the continuous PA data stream. The PA features were measured by the number of occurrences of the multiples of g-force (1 g, 2 g, and 3 g) in each 1-hour time window. One hour was further divided into 7200 time intervals of 500 milliseconds each. Within each 500-millisecond period, the AiCare platform detected whether the minimum level of acceleration (1 g) had occurred. If yes, it increased the 1-g value by 1 count. Therefore, on an hourly basis, a restless user could potentially accumulate up to 7200 values of 1 g. The same detection and computational logic applied to 2-g and 3-g values. The directionless g-force was an aggregation of the g-forces in 3 axes (directionless g-force = √ [g-force_x^2^ + g-force_y^2^ + g-force_z^2^]). This trial used the initial 72-hour period to build nonrisk baseline data and generated an alert when any deviation occurred, which indicated worsening health condition. The platform was able to detect the precursors of rehospitalization.

**Figure 1:**
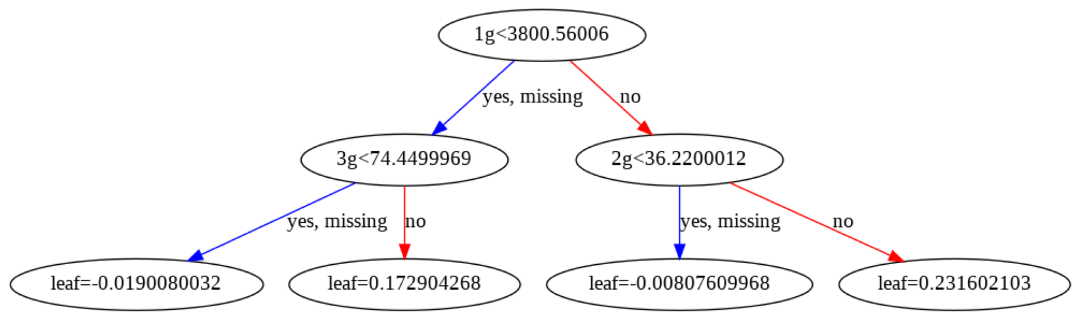
Sample decision tree.

A decision tree ensemble–based multiclass classification approach was used to predict no risk, mild risk, and risk. A maximum tree depth of 3 levels was deployed. The intrinsic graph of the decision tree facilitated the explainability of the model. Figure 1 presents a sample decision tree from our model.

This decision tree visualization provides insight into the gradient boosting process. Figure 1 illustrates the importance and data coverage of each input feature (1 g, 2 g, and 3 g) and the decision-making process. We chose cross-entropy–based softprob objective (the loss function in the first term of the training objective equation presented after this paragraph) to predict the probabilities of 3 categories in the risk profile. Because of the tendency of the decision tree to bisect the data space and to overfit the training data when classes are not well separated, we introduced a regularization term to balance the bias-variance trade-off (the second term of the training objective equation presented after this paragraph).



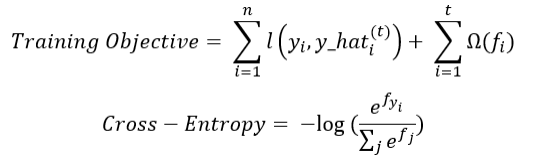



**(1)**

To reduce false-positive results, we further enhanced the platform with the clinician’s cognitive decision-based alerts. The AI model was trained continuously with the patients’ up-to-date PA data. Besides the AI model (Figure 1), the AiCare prediction platform was designed for higher scalability. The inbound data pipeline supports open APIs that are hardware agnostic and integrate with the PA features collected from different hardware devices. The outbound patients’ predictive risk profiles were streamed to various clinical applications to support broad clinical use cases. The ACT-I trial showed early signs of clinical efficacy with this low-cost noninvasive approach, enabling further scalability.

### Wireless Protocol

Considerations regarding the requirements of data collection, long-term use, power consumption, wireless transmission distance, legal radio frequency, home use, popularity, and cost led to choosing BLE as the optimal protocol for home care indoor use. The iPhone was connected to the cloud server via the standard wireless or cellular protocol.

### ID System and Data Collection

The personalized data were anonymized using an internally specified ID system for data collection. A BLE media access control address for each band enabled this functionality. The patients wore the WD (a battery-powered smart band [Rockband; AiCare Corp]) at all times to enable continuous data collection (the Rockband has a battery life of 45 days for continuous use).

### AiCare Technology

The AiCare platform enabled data collection and real-time analysis as described herein. The technology consisted of the following: (1) the low-cost and water-resistant Rockband with a battery life of 45 days for continuous use, (2) iPhone connectivity, (3) a cloud-enabled HIPAA-compliant AWS server, (4) open API architecture, (5) an ML and XGBoost interface, (6) an iPhone app, and (7) an AI-enabled COVID-19–specific questionnaire. This comprehensive platform was deployed in an iOS environment to analyze the patients’ PA data. We assessed PA by a triaxial accelerometer, which provides the optimal solution between technological complexity and reliable measurement of PA. This service was designed to ensure a smart, safe, and secure environment enabled with real-time, intelligent, and timely tracking, detection, and analysis to promote a healthy and independent lifestyle for discharged patients.

### Data Collection and Analysis

We used the Rockband for data collection. First, we defined the moving average (MA) of PA. The visualization of time series data obtained from AiCare’s platform allowed us to (1) identify changes in energy level (EL) and movement percentage, (2) establish a personalized baseline for each discharged patient, and (3) understand the data trend to predict any deviation in daily activity pattern.

### The MA of PA

The MA method is widely used to smooth out time series data by calculating the average values for a chosen period [4,12]. In this study, we used simple MA (SMA) to avoid the noisy measurements of the EL and movement percentage feature. Each data point was calculated using SMA in time series data and weighted equally. There was no need to set any weighting parameters such as the weighted or exponential MA method to generate SMA EL or movement percentages parameter. The SMA formula was defined as follows:



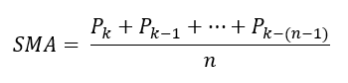



**(2)**

where *P_k_* represented the data point at time *k*, and *n* was the chosen number of data points. A longer-term SMA was less sensitive in reflecting the change in data movement compared with a shorter-term SMA, which was used to highlight the major trends in time series data. A shorter-term MA was relatively faster to react to changes in trend, which was beneficial to applications that required a QR code. The adjustment of the value *n* in the equation measured the different effects of trend analysis. The specific features and the definitions of PA are outlined in Textbox 1.

**Studied features and definitions for physical activity patterns.**

- Daytime energy level (EL): the EL obtained during the daytime period
- Nighttime EL: the EL obtained during the nighttime period
- Daily EL difference (ELD): the difference between daytime EL and nighttime EL
- Normalized ELD: the daily ELD in percentage values
- Daytime active percentage (AP): 100% minus daytime resting percentage (RP)
- Daytime RP: the percentage of zero movements during the daytime period
- Nighttime AP: 100% minus nighttime RP
- Nighttime RP: the percentage of zero movements during the nighttime period
- Daily active percentage difference: the difference between daytime AP and nighttime AP

### Kinetic EL

In this study, the estimation of kinetic energy was used to describe the EL of PA. The original formula of kinetic energy is as follows:

*Kinetic energy* = ½ × *mass* × *velocity*^2^ = ½ × *mass* × (*acceleration* × Δ*t*)^2^

**(3)**

The unit of kinetic energy is the joule (1 joule=1 kg m^2^/s^2^). To obtain a directionless measurement of acceleration from the accelerometer embedded in the Rockband, signal vector magnitude (SVM) was applied to calculate the overall magnitude of acceleration [13]:



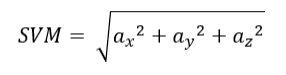



**(4)**

where *a_x_*, *a_y_*, and *a_z_* are the acceleration values from the triaxial accelerometer. In this system, the sampling rate of the accelerometer (Δt) is fixed and is equal to 50 Hz. The formula of kinetic energy can be rewritten as follows:

*Kinetic energy* = (½ × *mass* × Δ*t*^2^) × *acceleration*^2^ = *Constant* × *SVM*^2^

**(5)**

For each individual, the constant portion of this equation would be the same at any given time. Therefore, kinetic energy could be defined as SVM^2^ (m^2^/s^2^/kg). As a result, the estimation of total EL from time 0 to time n is defined as follows:

Total energy level = SVM2(t1) + SVM2(t2) + ... + SVM2(tn)

**(6)**

After obtaining the estimated wake-up time and resting time from the cloud platform, we can calculate the total energy expenditure during the daytime period and nighttime period, respectively.

The daytime period is equal to the time between wake-up and resting times on the same day, and the nighttime period is equal to the time between resting time and subsequent wake-up time on the following day. We used daytime EL to estimate the total intensity of all PAs that happened during the daytime period and nighttime EL to represent the total intensity of all PAs that happened during the resting period. In addition, the daily EL difference (ELD) has been used to evaluate the daily PA changes:

Daily energy level difference = ELDaytime – ELNighttime

**(7)**

A positive value of daily ELD indicates that daytime EL is greater than nighttime EL. It may represent that an individual is active during the day or inactive (sleeps well) during the night, which is a healthy PA pattern. A negative value of daily ELD can be obtained when nighttime EL is greater than daytime EL. High nighttime EL may represent disrupted sleep patterns, and thus movements can be detected by the Rockband at night. A negative EL difference also means that the observed individual is inactive during the day. To compare the change in individual ELD, normalization has to be performed to convert the absolute values of ELD into the percentage of ELD, which is defined by the following equations:



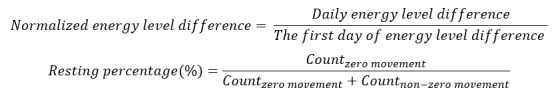



**(8)**

*Active percentage* (%) = 100% – *resting percentage* (%)

**(9)**

Daily active percentage difference (%) = Active percentageDaytime – Active percentageNighttime

**(10)**

### ML Algorithm for PA Analysis

**Figure 2:**
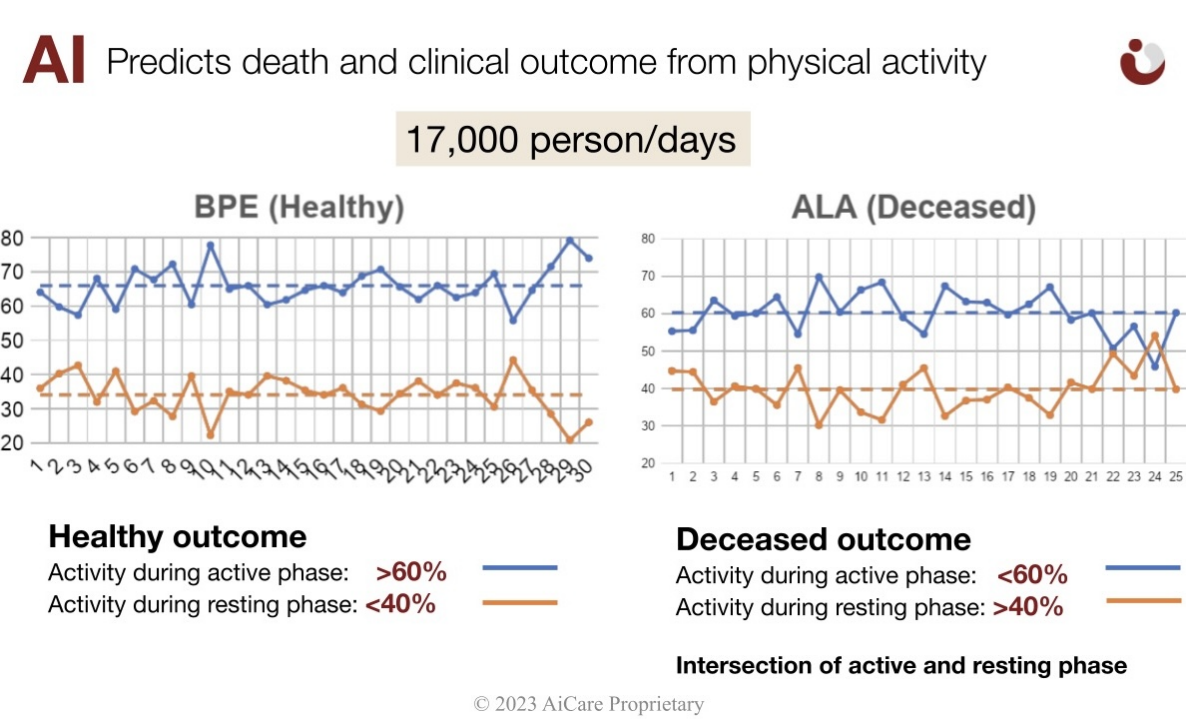
Data for clinical prediction. (A) Healthy outcome (physical activity [PA] ratio of active and resting phase: >60/40 ratio). (B) Deteriorating (deceased) outcome (PA ratio of active and resting phase: <60/40 ratio). ALA and BPE are the anonymized names of the patients.

Three key features—daily ELD, normalized ELD, and daily active percentage difference—were used to create the algorithm to predict the possible clinical worsening of discharged patients who demonstrate specific PA patterns. A risk alert was generated when the values of the features that were lower than a specific threshold were detected. The collected data correlated with the detailed measurement of the patients’ PA. We assessed the patients’ quality of recovery through accurate measurements of their activities while they were awake, while performing various activities of daily living (ADL) or participating in PA, and while resting. Multiple layers of big data analytics, data mining algorithms, and ML methods were tested. Specifically, we applied the XGBoost ML algorithm. Our solution refined the predictive capability by using the individual PA differences during the active phase (walking, standing, or sitting) versus the resting phase (lying down). XGBoost enhanced the predictive accuracy of healthy recovery versus deterioration at home and determined the need to contact health care professionals. XGBoost distinguished itself from other gradient boost learning methods by using clever penalization of trees, proportional shrinking of leaf nodes, Newton boosting, extra randomization parameter, and the implementation of single distributed systems. These features enabled efficient ML classification of the real-time monitoring of PA to refine the patients’ risk assessment. XGBoost distributed the feed-forward module of PA. The integration started with the PA module of 3 physical acceleration features (1 g, 2 g, and 3 g). The penalty-based system determined the initial risk profiling by PA weight and retrained in a stage-agnostic way to determine the features and penalties to enhance the weight from PA. The final stage repeats the same cycle as stage 2 and provides each patient’s final weight and risk score. As the individual stage is algorithm agnostic, this method provides randomized nonbiased Newtonian analysis. The training data set was achieved by our solution by predicting the clinical outcome based on the individual PA differences during the active phase (walking, standing, or sitting) versus the resting phase (lying down). On the basis of >17,000 person-day data points of 36 participants (unpublished data), we predicted healthy recovery versus death in skilled nursing facility residents based on the PA data and analysis (Figure 2). Using this training data set, our XGBoost algorithm was designed to detect deterioration in the health condition of the discharged patients to generate a risk alert, which suggested the need for early medical intervention by contacting health care professionals, and prevent hospital readmission.

### Statistics and the AUC-ROC Curve

The ACT-I trial was an observational study, and we performed a correlational analysis between PA and clinical outcomes by using the AI model. The population consisted of 249 patients discharged from Stanford Hospital general cardiology and heart failure services. The measurement units relate to 36 (14.5%) of the 249 patients who completed a 28-day analysis. The response is the clinical outcome, and the factor is a comprehensive profile of PA from the patients. The choice of model is XGBoost. XGBoost-generated graph is a commonly used graph that summarizes the performance of a classifier over all possible thresholds. It is generated by plotting the true-positive rate (y-axis) against the false-positive rate (x-axis) as the threshold for assigning observations to a given class. AUC measures the entire 2D area under the ROC curve. The maximum value it can reach is 1; generally, the greater the value, the better the performance of the model.

ROC analysis was used to evaluate a classifier’s prediction performance in biological and medical applications. Each data point in the ROC curve comprises a pair: true-positive rate (sensitivity) and false-positive rate (1−specificity), generated by a discrete classifier with a specific threshold [14]. This study used several periods of MA from 3 days to produce all ROC points in the ROC space. Considering the effect of the imbalanced data set, meaning that the number of healthy discharged patients is greater than the number of discharged patients classified as deteriorating, we also used the recall-precision curve to evaluate the performance of the proposed algorithms. In the recall-precision curve space, the x-axis and y-axis represent the recall values and precision values, respectively, calculated from the different thresholds.

### User Interface

**Figure 3:**
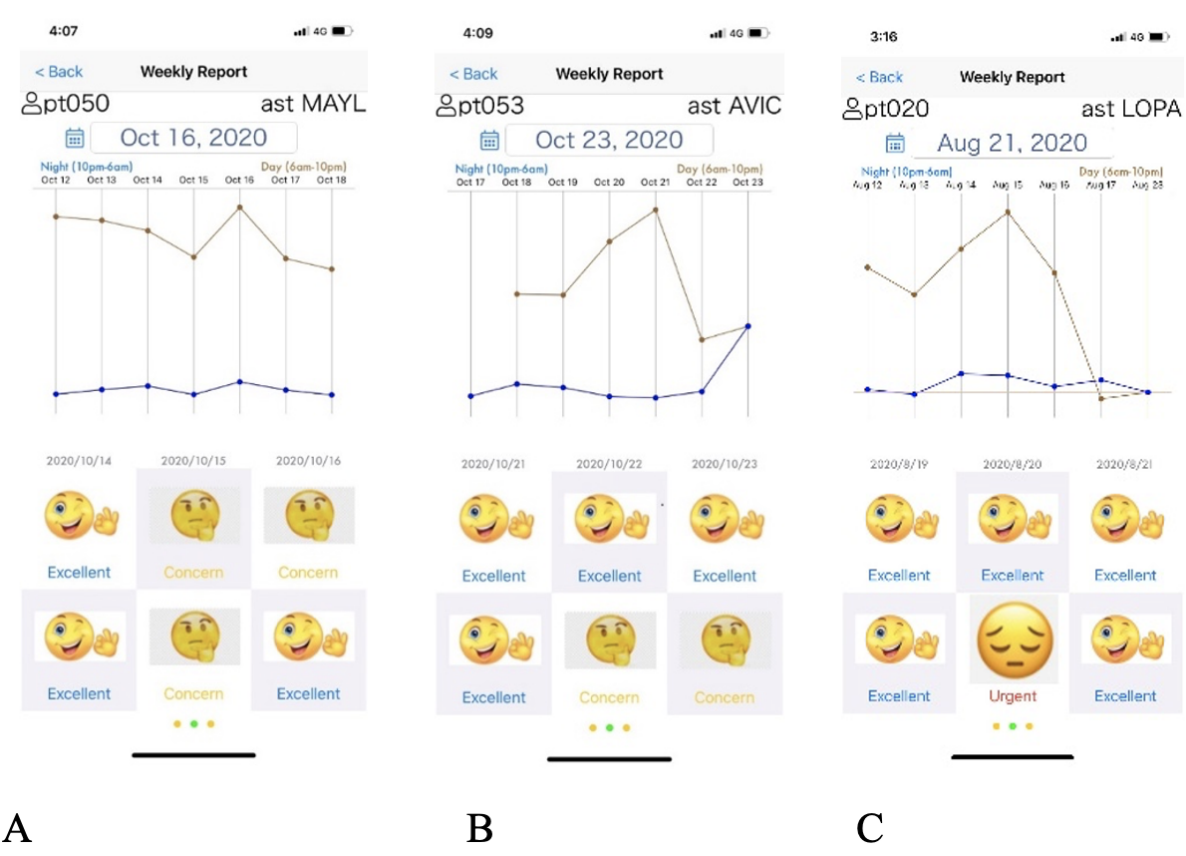
Graphic user interface for alert notifications in 3 representative patients. MAYL, AVIC, and LOPA are also anonymized names of patients.The descriptor and smiley face emoji indicate (A) “Excellent” health followed by the descriptor “Concern” and pensive face emoji for mild risk, (B) “Excellent” health followed by the descriptor “Concern” and pensive face emoji for mild risk, and (C) “Excellent” health followed by the descriptor “Urgent” and sad face emoji for indication of risk. ast: assistance.

On the weekly report, we displayed the line chart, which kept track of the patient’s activity level, and at the bottom, we generated a user-friendly emoji to report the health condition measured by our algorithm every 12 hours. The descriptor “Excellent” and a smiley face emoji indicate a healthy and normal pattern. The descriptor “Concern” and a pensive face emoji mean that there was an unusual pattern, indicating that the patients should be aware of the possible worsening of their clinical condition. Finally, the descriptor “Urgent” and a sad face emoji signify an unhealthy signal from the patient’s PA pattern. This prediction suggests that the patients should contact their health care professionals (Figure 3).

## Results

### Patient Enrollment

We screened 249 patients discharged from Stanford Hospital general cardiology and heart failure services. Of these 249 patients, 52 (20.9%) were enrolled, and 36 (14.5%) completed a 28-day analysis. The reasons for noncompletion were as follows: (1) withdrawal from study (10/16, 63%), (2) battery failure (4/16, 25%), and (3) early readmission (2/16, 13%). Of the 36 patients, 30 (83%) responded to the ADL questionnaire and 30 (83%) responded to the satisfaction questionnaire.

### Prediction

**Figure 4:**
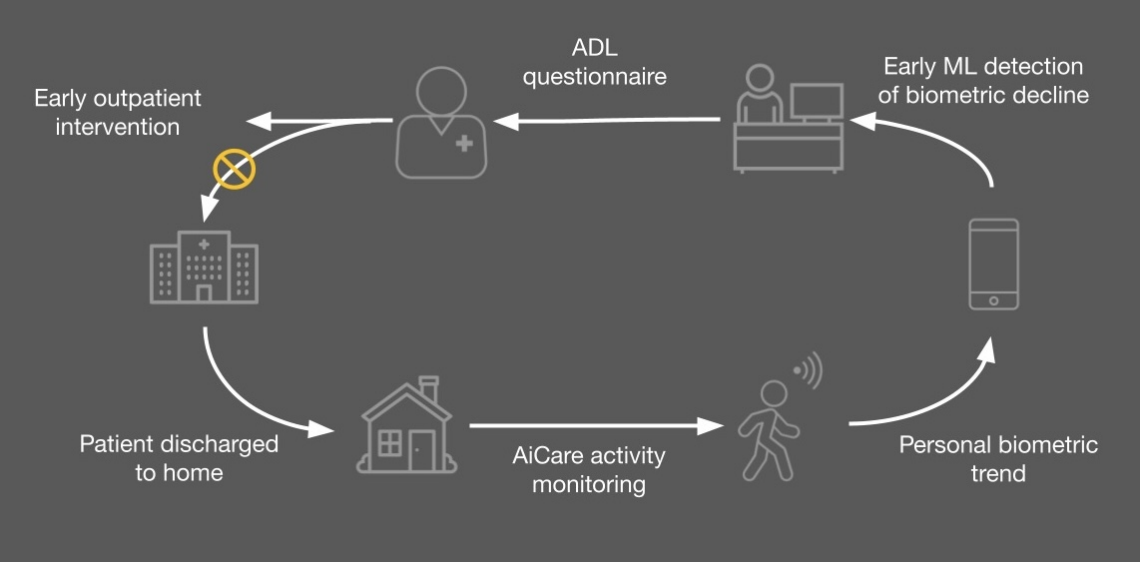
Patient flow. ADL: activities of daily living; ML: machine learning.

Our data demonstrated a robust prediction system to forecast the worsening clinical outcomes of these patients based on their PA data, achieving a sensitivity of 87% and a specificity of 79%. On the basis of real-time assessment of PA, our technology offered clinically reliable predictions regarding the discharged patients who would need to contact their health care professionals or caretakers to report their worsening clinical condition. This capability allowed early intervention to prevent further deterioration of these patients (Figure 4) [10].

After the patient’s discharge to home, the AiCare platform is deployed to the patient via the Rockband and iPhone. PA data and trend are displayed on the iPhone app. If there is any negative change in PA, the patient is contacted for a clinical evaluation. The ADL questionnaire is administered to the patient to see whether there is any correlation.

Our solution provided a robust low-cost technology to measure PA and predict clinical outcomes. Our platform also included the nudge technology for an AI-enabled questionnaire. This platform was designed to deliver a seamless, low-cost, and user-friendly environment for the remote monitoring of discharged patients at home to empower the patients and family members. This study analyzed the predictive capability of our platform as described in Textboxes 2-4.

The clinical diagnoses were categorized into true positive, true negative, false positive, and false negative as shown in Textbox 5.

The technical parameters were achieved and categorized into true positive (TP), true negative (TN), false positive (FP), and false negative (FN) as shown in Textbox 6.

Questionnaires were also administered at the time of risk alert as well as at the completion of the study (Textbox 7). The ADL questionnaire was administered to augment the PA data. The findings demonstrated modest correlation with the predictive capability. The patients whose condition deteriorated (true-positive group) showed the lowest function in terms of ADL, whereas those who remained stable showed higher response scores (true-negative group). However, there were low ADL scores in the false-positive group and high ADL scores in the false-negative group. The responses to the satisfaction questionnaire demonstrated that this platform was well received. The majority of the users stated that they would recommend the technology to others.

**Duration to risk prediction and intervention categorized into true positive (TP), true negative (TN), false positive (FP), and false negative (FN).**

- Mean number of days from discharge to risk prediction: TP=9 (SD 4); TN=none; FP=7 (SD 3); and FN=none
- Mean number of days from risk prediction to patient-initiated contact of health care professional: TP=5 (SD 2); TN=none; FP=2 (SD 2); and FN=none
- Total activity (1 g, 2 g, and 3 g per patient): TP=33,351 (SD 15,774); TN=38,998 (SD 19,062); FP=43,430 (SD 16,638); and FN=30,714 (SD 16,998)

**Formulas of area under the receiver operating characteristic curve metrics (TP=true positive, TN=true negative, FP=false positive, and FN=false negative).**

**Accuracy:**

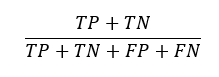

**Precision and positive predictive values:**

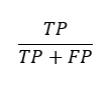

**Sensitivity, recall, or true-positive rate (TPR):**

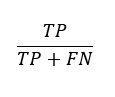

**Specificity or true-negative rate:**

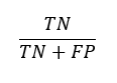

**Negative predictive values (NPV):**

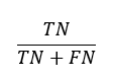

**False-positive rate (FPR)= 1 − specificity:**

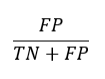

**Calculated values for the area under the receiver operating characteristic curve.**

- Sensitivity: 90.01%
- Specificity: 81.55%
- Positive predictive value: 77.1%
- Negative predictive value: 92.3%
- Accuracy: 85%
- False-positive rate: 18.45%

**Clinical diagnosis and prediction data.**

**True positive (n=17)**

- Heart failure (n=9)
- Arrhythmia (n=6)
- Atrial (n=5)
- Ventricular (n=1)
- Device (n=1)
- Ischemia (n=1)

**True negative (n=9)**

- Arrhythmia (n=6)
- Atrial (n=4)
- Ventricular (n=2)
- Ischemia (n=2)
- Heart failure (n=1)

**False positive (n=11)**

- Arrhythmia (n=5)
- Atrial (n=3)
- Ventricular (n=2)
- Heart failure (n=2)
- Ischemia (n=2)
- Pulmonary hypertension (n=2)

**False negative (n=2)**

- Arrhythmia, ventricular (n=1)
- Pericarditis (n=1)

**Technical findings.**

- Signal loss hours per patient: TP=79; TN=74; FP=71; and FN=60
- Battery life per patient (d): TP=34; TN=29; FP=32; and FN=19
- Early replacement of the Rockband (number of patients): TP=1; TN=4; FP=0; and FN=1

**Response to the 7-question activities of daily living (ADL) questionnaire (7/7, 100%: highest function; n=30).**

- True positive: 5.5 (positive ADL engagement)
- True negative: 6.25
- False positive: 4.7
- False negative: 7

## Discussion

### Principal Findings

We screened 249 patients discharged from Stanford Hospital general cardiology and heart failure services. Of these 249 patients, 52 (20.9%) were enrolled, and 36 (14.5%) completed a 28-day analysis looking into the correlation between PA and clinical outcome. Using our XGBoost model, we plotted the true-positive rates against false-positive rates, which helped us to generate the AUC-ROC curve and calculate the 2D AUC value to determine the performance of the model. Our data demonstrated a robust prediction system to forecast the worsening clinical outcomes of these patients based on their PA data, achieving a sensitivity of 87% and a specificity of 79%.

This innovative platform enables low-cost, robust, and precise PA tracking of patients discharged from hospital to predict stable versus unstable clinical recovery, using a WD and an iPhone. This technology is powered by our algorithms, individualized big data, personalized behavior- and function-specific web-based software, and intelligent ML analytics. This comprehensive platform offers an effective convergence of eHealth, AI, and telemedicine technology over internet-enabled mobile devices to leverage the economical, low-cost, and pervasive internet technology and, potentially, may address the socioeconomic divide seen today. Patient care at home by family members or by the individual patient is personalized by AI for maximum safety. This technology will fill an important gap in telemedicine through the use of user-friendly, real-time, and 24/7 remote monitoring for clinical outcome prediction. Patients in transition who are discharged from the hospital, emergency department, or urgent care clinic will benefit from this technology, which can monitor their progress and predict clinical deterioration to enable early intervention for successful recovery at home.

This ACT-I trial used monitoring technology to measure in-home activity and predict clinical outcomes. Although many innovative technologies claim accurate measurements of vital signs, there is no platform with proper validation of clinical outcome prediction data. A wide range of longitudinal studies to demonstrate the effectiveness of remote monitoring technology have been performed [10,15]. Most research was conducted within the area of passive infrared motion sensor technology, followed by research on body-worn sensors. Although the research into the use of monitoring technologies has been extensive, most studies only focused on demonstrating the functionality of the proposed monitoring technology by simulating activities in a laboratory setting or on an existing data set. As a result, the functionality of most systems has only been demonstrated in general terms or mechanical accuracy, sensitivity, and specificity. The long-term clinical effects of using monitoring technology are less well studied; for instance, in a meta-analysis on ambient sensors for older adult care, 25 of the 141 studies were pilot studies, with 11 focusing on the use of passive infrared motion sensor technology and 10 on the use of multicomponent monitoring technology. Study durations ranged from 3 weeks to 3 years [16]; only 4 studies were longitudinal, including 1 randomized controlled trial and 1 implementation study [17]; and all focused on the use of motion sensor technology. WDs have evolved from merely telling time to encompassing ubiquitous computing applications, miniaturized sensors, and wearable computer technology. Fitbit released its first wearable watch in 2009 and focused on activity tracking. During the ensuing years, smartwatches became common technology products manufactured by electronics companies. These developments led to a set of design guidelines for wearability and WDs that make tracking PA a much more attractive target for discharged patients [9].

PA is one of the major determinants for overall clinical outcomes in chronic diseases, including diabetes, hypertension, and heart disease, as well as mental health issues [17]. Reaching a sufficient level of PA could reduce the risk of cardiovascular disease (CVD), type 2 diabetes, obesity, depression, and anxiety [18-20]. PA even plays an important role in cancer prevention at specific sites, including breast and colon cancers [16]. In 2017, the Centers for Disease Control and Prevention advocated adding PA as 1 of the 4 vital signs [20]. Despite these efforts, automated clinical outcome prediction systems do not exist. There is a need for the accurate prediction of morbidity and mortality, particularly among older adults who are most frequently readmitted to the hospital. Patients with chronic diseases, cardiometabolic syndrome, and dementia are often underserved, growing in numbers, incurring higher costs to our society, and becoming increasingly vulnerable. Our large data set obtained from a skilled nursing facility and consisting of >17,000 person-day data points (408,000 person-hour data points of 36 patients captured over 2 years), demonstrated a high correlation of PA analysis among the residents who survived versus those who died. Using this as our training data set, we were able to identify with high accuracy patients who experienced stable recovery versus those who experienced unstable recovery during the most vulnerable 1-month posthospital discharge period.

The evidence-based management of CVDs requires substantial amounts of resources, including advanced therapeutics, complex diagnostics, and sophisticated clinical trials. However, the reliable prediction of clinical outcomes after hospital discharge has presented some challenges [11]. In response, various approaches using ML models such as artificial neural network, decision tree, support vector machine, and naïve Bayes have been attempted to predict clinical outcomes, taking into account steps, vital signs, medical conditions, and demographic information [11]. In one of the studies conducted on arrhythmic sudden cardiac death, a deep learning technology approach termed Survival Study of Cardiac Arrhythmia Risk was developed to predict risk for 156 patients with ischemic heart disease. In this model, cardiac magnetic resonance images and covariate data such as demographics, risk factors, electrocardiogram (ECG) measurements, medication use, and outcomes were used as inputs for the 2 branches, where 1 branch is used to visualize the heart’s 3D ventricular geometry, and the other is used to extract arrhythmic sudden cardiac death risk–related imaging features from the cardiac magnetic resonance images. All these data were then used to create a survival curve individualized for each patient with accurate predictions for up to 10 years. However, the limitations of this study include an inability to account for competing risks for the same symptoms and covariates that were not exhaustive or fully inclusive [17]. In another instance, regression and convolutional neural network models were used to predict CVD risk for women. As CVDs are the primary cause of death in women, with evidence of sex bias in the diagnosis of CVDs , the exploration of screening factors for risk detection has never been more urgent. This study assessed the critical risk–screening opportunities offered to women and how the integration of AI can greatly benefit health care providers in interpreting data on women [17,21]. AI may propel the analysis of patient data into meaningful interpretations of patient health, providing health care providers with an additional layer of guidance for patient management plans. After patient discharge, ML is still viable in assisting with remote health monitoring through systems such as Wanda-CVD, which uses patients’ blood pressure and BMI measurements as well as their low-density lipoprotein and high-density lipoprotein cholesterol levels to coach them and improve their risk factors for CVD. However, using limited inputs such as blood pressure readings and cholesterol levels may not be entirely meaningful. In a previous study, only less than half of the predictions based solely on cholesterol levels and BMI measurements were correct [8,22].

In contrast, our XGBoost ML model enhanced the accuracy of predicting stable recovery versus clinical deterioration after discharge from the hospital. Specifically, XGBoost performs self-adaptive feature selection and prioritization among our data dimensions, mitigates the risk of overfitting by controlling the complexity of the trees with penalization on leaf nodes to cope with the high-frequency nature of our temporal data set, uses a Newton boosting algorithm to better learn the tree structures, and decorrelates the individual trees with a randomization parameter to reduce the bias and variance of the model. Compared with the existing approach, our AI-enabled solution is unique in the following ways. First, our real-time PA-based algorithm enables risk prediction during the critical 1-month posthospital discharge period, whereas most of the other research on risk prediction focused on a much longer time frame of 5 to 10 years. Our approach allows a shift to early intervention and the prevention of clinical deterioration during the postdischarge period. Second, our training data set for the ACT-I clinical trial consisted of a large number of data sets obtained during a long follow-up period. Our data covered a 2-year duration, which enabled a longitudinal follow-up for a personalized benchmark to conduct individualized analysis and risk prediction. Third and last, our low-cost at-home patient onboarding process did not rely on complex hardware such as imaging or remote ECG equipment. The Rockband WD was low cost, maintenance free, and disposable. Our hardware-agnostic AI framework demonstrated highly and easily adaptable features using our simple WD.

### Limitations

Although the majority of the patients were satisfied with our platform, there were some compliance issues related to the use of the WD. Furthermore, the measurement of PA only may not provide a comprehensive assessment and prediction of an individual patient’s clinical condition. Our future trial, ACT-II, will expand on the ACT-I trial’s limitations by improving the specificity (false positive) rate of the ACT-I trial by evaluating the efficacy of an augmented XGBoost algorithm. We will use an Apple Watch to complement PA measurements by also monitoring HR, HR variability, ECG, oxygen saturation, blood pressure (separate blood pressure measurement device), clinical data, and genomics to better identify stable versus unstable recovery. Our novel platform in an iOS environment will enable the capture of multidimensional real-time data to enhance patients’ awareness of their clinical condition and health care professionals’ guidance of patient management. We will investigate the feasibility of this platform, consisting of an Apple Watch, an iPhone, an XGBoost interface, and a HIPAA-compliant AWS environment, to monitor the dynamic biometric data, predict patients’ clinical outcome, and improve patient compliance.

### Conclusions

The ACT-I trial demonstrated a critical proof of concept of the Rockband WD to enable real-time analysis of patients’ PA data remotely. We developed a cloud-enabled XGBoost algorithm and intelligent sensor technology to enable precision home health care. The XGBoost algorithm quantified, integrated, and predicted the pattern of each patient’s outcome seamlessly with high accuracy, precision, and recall. The Rockband cloud backend personalized the big data for behavior- and function-specific interactive software, and ML analytics allowed a comprehensive platform to converge eHealth, AI, and telemedicine technology. Our internet-enabled mobile devices leveraged the economical, low-cost, and pervasive technology to personalize health care by enabling prevention and early intervention through the real-life clinical implementation of mobile device technology and AI. Our approach developed, tested, and disseminated the next generation of health care strategy by focusing on precision health, using diagnostic information collected in real time from patients’ PA data while they were recovering at home. Our XGBoost algorithm enabled this scalable, portable, and distributed processing framework. This novel technology will introduce a nascent approach to patient care to redefine clinical practice by predicting patient outcome based on a comprehensive analysis of behavioral phenotype. This real-time risk monitoring and clinical outcome prediction platform will advance the future of remote patient care.

## Abbreviations

ACT-I: Activity Assessment of Patients Discharged from Hospital-I
ADL: activities of daily living
AI: artificial intelligence
API: application programming interface
AUC: area under the curve
AUC-ROC: area under the receiver operating characteristic curve
AWS: Amazon Web Services
BLE: Bluetooth low energy
CVD: cardiovascular disease
ECG: electrocardiogram
EL: energy level
ELD: energy level difference
HIPAA: Health Insurance Portability and Accountability Act
HR: heart rate
HRRP: Hospital Readmissions Reduction Program
MA: moving average
ML: machine learning
PA: physical activity
ROC: receiver operating characteristic
SMA: simple moving average
SVM: signal vector magnitude
WD: wearable device
XGBoost: extreme gradient boosting

## Data Availability

We hope to submit our data to *JMIR Data*. However, we obtained the informed consent to release their data in our repository; therefore, this may not be possible.
