# Correction: Cloud-Based Machine Learning Platform to Predict Clinical Outcomes at Home for Patients With Cardiovascular Conditions Discharged From Hospital: Clinical Trial

**DOI:** 10.2196/68825

**Published:** 2024-12-10

**Authors:** Phillip C Yang, Alokkumar Jha, William Xu, Zitao Song, Patrick Jamp, Jeffrey J Teuteberg

**Affiliations:** 1 Stanford University School of Medicine Palo Alto, CA United States; 2 AiCare Corporation San Jose, CA United States; 3 Emory University Atlanta, GA United States; 4 North Carolina State University Raleigh, NC United States; 5 Electrical Engineering University of California Los Angeles, Mountain View, CA United States

In “Cloud-Based Machine Learning Platform to Predict Clinical Outcomes at Home for Patients With Cardiovascular Conditions Discharged From Hospital: Clinical Trial” (JMIR Cardio 2024;8:e45130) the authors made one addition to their affiliations.

The following affiliation has been added to authors PCY, AJ, WX, ZS, and PJ:

AiCare Corporation, San Jose, CA United States

Therefore, the revised authors' affiliations are as follows:

*Phillip C Yang^1, 2^, MD; Alokkumar Jha^1, 2^, PhD; William Xu^2, 3^, BSc; Zitao Song^2, 4^, MSc; Patrick Jamp^2, 5^, BSc; Jeffrey J Teuteberg^1^, MD*

1. Stanford University School of Medicine, Palo Alto, CA, United States

2. AiCare Corporation, San Jose, CA United States

3. Emory University, Atlanta, GA, United States

4. North Carolina State University, Raleigh, NC, United States

5. Electrical Engineering, University of California, Los Angeles, Mountain View, CA, United States

The correction will appear in the online version of the paper on the JMIR Publications website on December 10, 2024, together with the publication of this correction notice. Because this was made after submission to PubMed, PubMed Central, and other full-text repositories, the corrected article has also been resubmitted to those repositories.

